# Piriform cortical glutamatergic and GABAergic neurons express coordinated plasticity for whisker-induced odor recall

**DOI:** 10.18632/oncotarget.21207

**Published:** 2017-09-23

**Authors:** Yahui Liu, Zilong Gao, Changfeng Chen, Bo Wen, Li Huang, Rongjing Ge, Shidi Zhao, Ruichen Fan, Jing Feng, Wei Lu, Liping Wang, Jin-Hui Wang

**Affiliations:** ^1^ Department of Pathophysiology, Bengbu Medical College, Bengbu 233000, China; ^2^ Institute of Biophysics, Chinese Academy of Sciences, Beijing 10010, China; ^3^ University of Chinese Academy of Sciences, Beijing 100101, China; ^4^ Shengzheng Institute of Advanced Technology, Chinese Academy of Sciences, Shenzhen 518055, China; ^5^ Qingdao University, School of Pharmacy, Qingdao 266021, China

**Keywords:** memory, glutamate GABA neuron, synapse, piriform cortex, plasticity homeostasis

## Abstract

Neural plasticity occurs in learning and memory. Coordinated plasticity at glutamatergic and GABAergic neurons during memory formation remains elusive, which we investigate in a mouse model of associative learning by cellular imaging and electrophysiology. Paired odor and whisker stimulations lead to whisker-induced olfaction response. In mice that express this cross-modal memory, the neurons in the piriform cortex are recruited to encode newly acquired whisker signal alongside innate odor signal, and their response patterns to these associated signals are different. There are emerged synaptic innervations from barrel cortical neurons to piriform cortical neurons from these mice. These results indicate the recruitment of associative memory cells in the piriform cortex after associative memory. In terms of the structural and functional plasticity at these associative memory cells in the piriform cortex, glutamatergic neurons and synapses are upregulated, GABAergic neurons and synapses are downregulated as well as their mutual innervations are refined in the coordinated manner. Therefore, the associated activations of sensory cortices triggered by their input signals induce the formation of their mutual synapse innervations, the recruitment of associative memory cells and the coordinated plasticity between the GABAergic and glutamatergic neurons, which work for associative memory cells to encode cross-modal associated signals in their integration, associative storage and distinguishable retrieval.

## INTRODUCTION

Neural plasticity is presumably associated to memory formation [1, 2]. For instance, excessive stimulation or deprivation from innate inputs induces the structural and functional plasticity of dendritic spines, excitatory synapses and neural circuits in their communicated sensory cortices [3–17]. Neural circuits include excitatory and inhibitory neurons. Their physiological interactions and balances are important for the brain to program neural codes that manage well-organized cognitions [18–20]. How the different types of neurons and synapses are recruited and refined coordinately for the storage and retrieval of new featured signals remains elusive [21–24].

Associative learning is a common way for information acquisition, and associative memory is essential for cognition [25, 26]. Classical conditioning as the typical form of associative learning has been used to study mechanisms underlying associative memory, in which animal behaviors in response to unconditioned stimulus can be induced by conditioned stimulus [27–29]. In this cross-modal reflex, the brain area to encode unconditioned signal may encode conditioned signal. Current reports show that paired whisker and odor stimulations lead to odorant-induced whisker motion and whisker-induced olfaction response, a reciprocal form of cross-modal associative memory. The neurons in barrel and piriform cortices are recruited to encode both whisker and odor signals, i.e., associative memory cells [21, 30–32]. As the whisker signal is new to the piriform cortex and the odor signal is new to the barrel cortex before associative learning, both cortices store the newly acquired signals, cross-modal memory. This model is useful to study the mechanisms underlying the memory of new information as well as to reveal the working principle of memory cells to accept, store and retrieve the newly acquired signals during associative memory [21, 24, 25, 31, 33].

To examine coordinated plasticity at associative memory cells including excitatory and inhibitory neurons in the piriform cortex for storing the newly acquired whisker signal and the innate odor signal, we trained mice by pairing whisker and odor stimulations to induce whisker-induced olfaction response. In our study, the mice whose cortical glutamatergic neurons were genetically labeled by yellow fluorescent protein and GABAergic cells were green fluorescent protein [24, 34] were used to analyze cell-specific mechanisms. Whether piriform cortical neurons were recruited to be associative memory cells for encoding odorant and whisker signals was examined by electrophysiological recording *in vivo*. The structural refinement of the glutamatergic and GABAergic neurons in the piriform cortex was assessed by confocal cellular imaging. Functional plasticity in the spike encoding and synaptic dynamics was assessed by whole-cell recording in the piriform cortical areas of brain slices. By these analyses, we aim to figure out how excitatory and inhibitory neurons are coordinately recruited and refined in order to integrate, encode and memorize associative signals.

## RESULTS

### Piriform cortex becomes to encode innate odor signal and new whisker signal after their association

**Figure 1:**
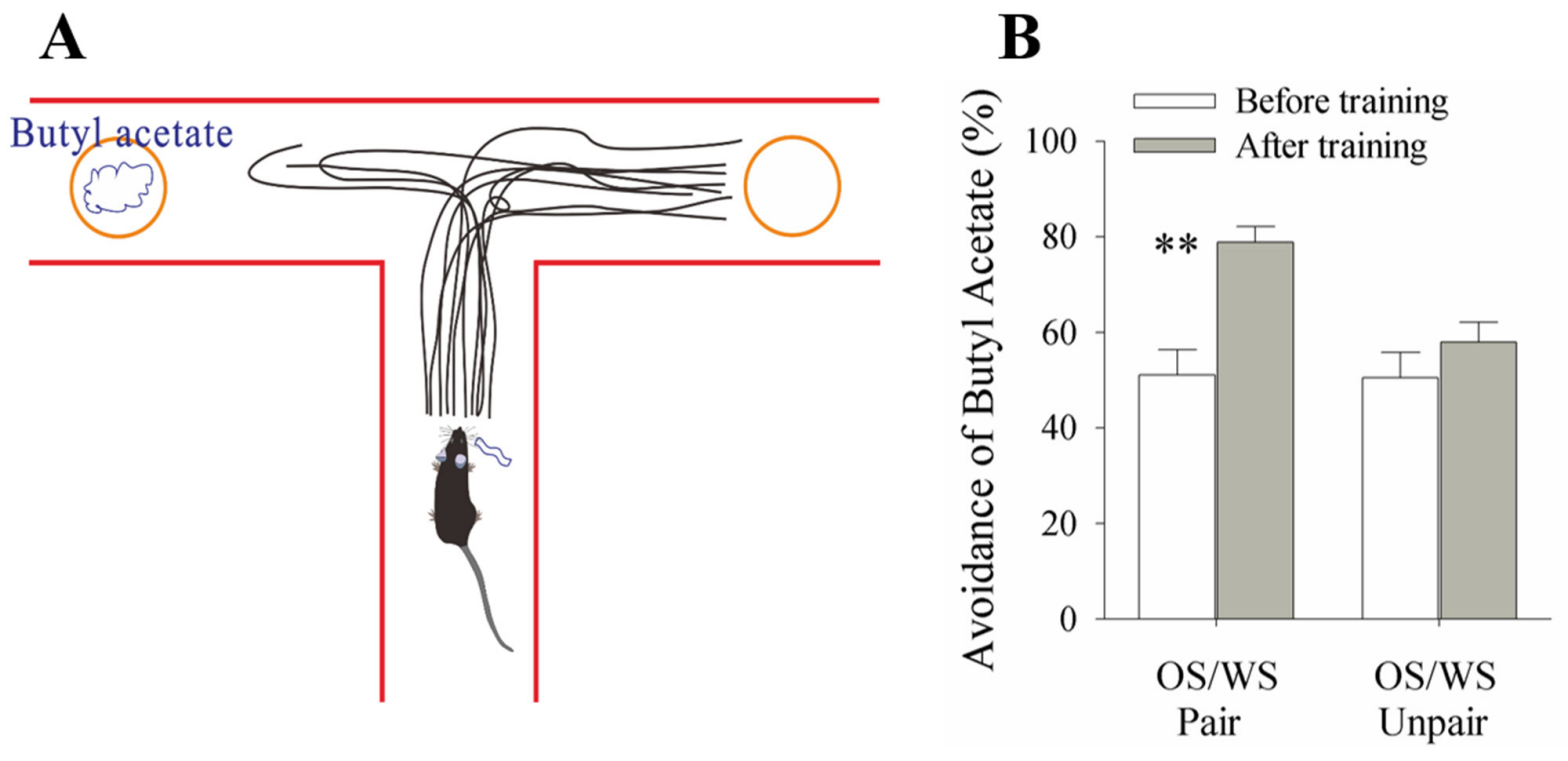
A simultaneous pairing of whisker stimulus (WS) and olfactory stimulus (OS) leads to whisker-induced olfaction responses WS and WS-test consisted of mechanical pulses at 5 Hz and an intensity of evoking whisker response. OS was butyl acetate pulse that sufficiently evoked olfactory bulb response. Stimulus durations were 20 seconds. **(A)** A mouse was placed in the central arm of “T” maze. The object coated with butyl acetate is placed in an arm, and the object without butyl acetate is placed in another arm. The distance from the objects to the center is set in a situation that the mice before training and in control are just unable to identify butyl acetate. This 50% selection rate indicates a minimal concentration of their olfactory sensitivity. While stimulating their whiskers, the recall of smelling butyl acetate drives the mice moving toward the control arm. The moving traces in CR-formation mice indicate their preference away from butyl acetate. **(B)** shows the percentage of selecting control arm versus butyl acetate in the groups of OS/WS-pair mice (left columns) and OS/WS-unpair control (right) before (white bars) and after trainings (grays). The high rate of selecting the control arm are observed in the OS/WS-paired mice with whisker-induced olfaction response (p<0.01, n=24; paired-test for comparisons before versus after training; and ANOVA for the comparison among groups).

The paradigm of associative learning was pairing whisker stimulus (WS) and odor stimulus (WS) in mice for ten days. In response to WS during the testing, the mice that have received OS/WS-pairing intend to move away from a T-maze arm that includes aversive butyl acetate in a rate of 78.8±2.3% (Figure 1, n=24), compared with no change in OS/WS-unpairing group (n=15). Their olfaction-related responses to the WS are thought to be a whisker-induced recall of olfactory signal, i.e., whisker-induced olfaction responses or conditional responses (CR). Because the distance between the central zone and the blockers is larger than 40 cm that the mice without OS/WS-pairing are unable to identify the source of odors [35], so that whisker-induced olfaction response is the outcome of the WS and OS association. This result reveals that whisker signal triggers the recall of odorant signal, i.e., cross-modal memory.

In terms of the mechanisms, we assumed that associative learning recruits the piriform cortex to encode the newly learnt whisker signal alongside innate olfactory signal. That is, the piriform cortex became the common area of odorant-induced olfaction response (unconditioned reflex) and whisker-induced olfaction response (conditioned reflex). The recruitment of associative memory neurons in piriform cortices of CR-formation mice was examined by recording local field potentials (LFP) *in vivo*.

**Figure 2:**
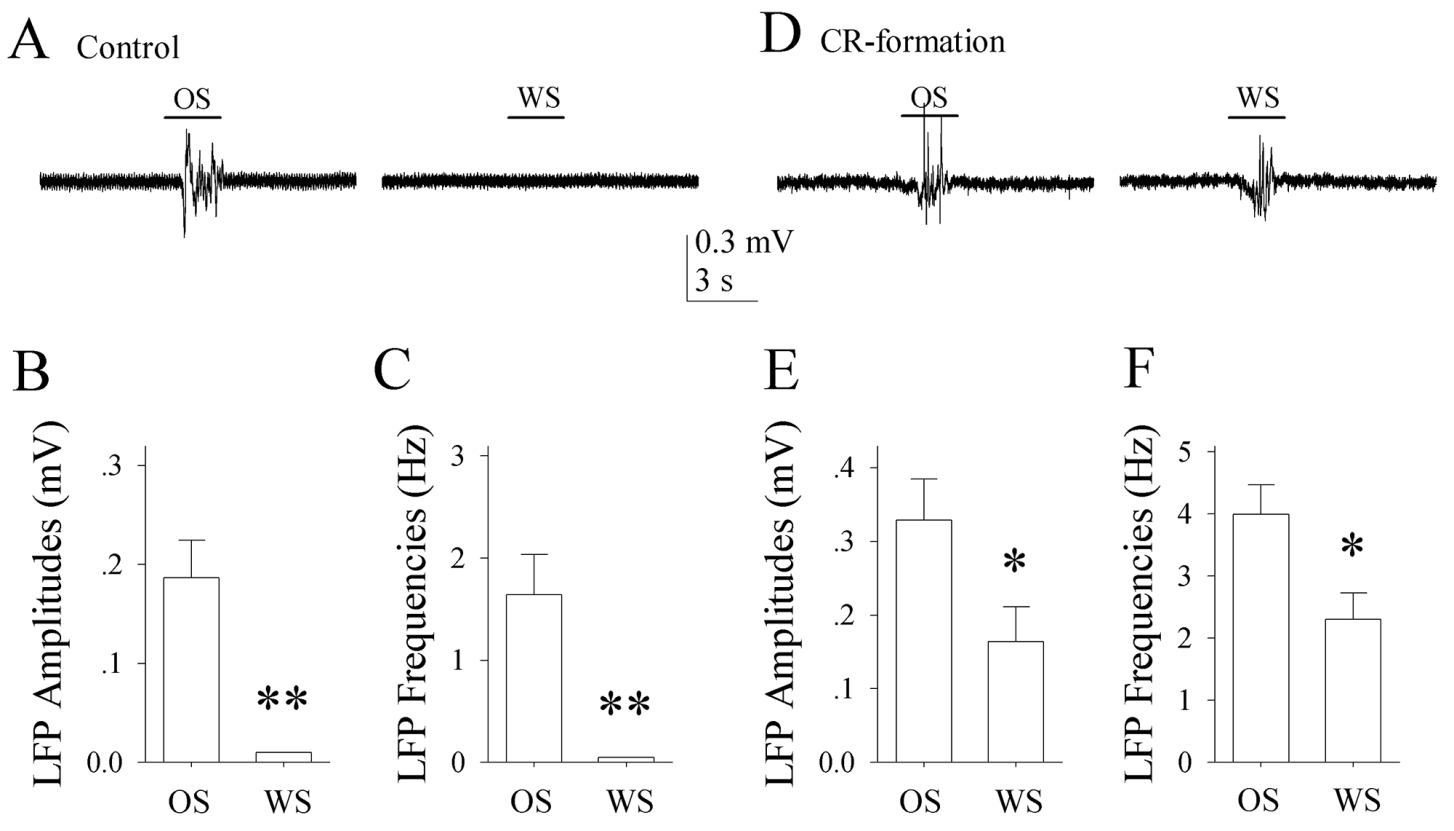
The neurons of the piriform cortices in CR-formation mice encode and distinguish OS and WS The neuronal activities were recorded by local field potential (LFP) *in vivo*. **(A)** shows that the neurons in the piriform cortex from a control mouse respond to OS (left trace), but not to WS (right). **(B)** shows LFP amplitudes recorded from the piriform cortex of control mice in response to OS and WS (p<0.001, n=14; one way ANOVA). **(C)** illustrates LFP frequencies recorded from the piriform cortex of control mice in response to OS and WS (p<0.001, n=14). **(D)** shows that the neurons of the piriform cortex from a CR-formation mouse respond to both OS (left trace) and WS (right). **(E)** shows LFP amplitudes recorded from the piriform cortex of CR-formation mice in response to OS and WS (p<0.05, n=14; one way ANOVA). **(F)** shows LFP frequencies recorded from the piriform cortex of CR-formation mice in response to OS and to WS (p<0.05, n=14). Calibration bars are 0.3 mV/3 seconds.

In control mice, the neurons in the piriform cortex respond to the OS, but not to the WS (Figure 2A-2C). In CR-formation mice, the piriform cortical neurons respond to the WS and the OS (Figure 2D). LFP amplitudes are 0.16±0.047 mV in response to the WS and 0.33±0.06 mV to the OS (Figure 2E; p<0.05, n=14; paired t-test). LFP frequencies are 2.31±0.42 Hz in response to the WS and 3.99±0.48 Hz in response to the OS (Figure 2F; p<0.05, n=14; paired t-test). It is noteworthy that piriform cortical neurons are significantly different in response to WS, but not different in response to OS before and after associative learning. The results indicate that the neurons in the piriform cortex are recruited to encode the newly learnt whisker signal alongside the innate odor signal for their integration and associative memory, i.e., associative memory cells. Their different responses to the WS and OS may underlie their recognitions during signal retrieval.

A recruitment of associative memory neurons in the piriform cortex that encode both whisker and odorant signals is hypothetically driven by coordinated plasticity at excitatory and inhibitory neurons and/or by mutual connections between the piriform and barrel cortices. These hypotheses are tested below.

### The connection from the barrel cortex to piriform cortex is established after associative learning

**Figure 3:**
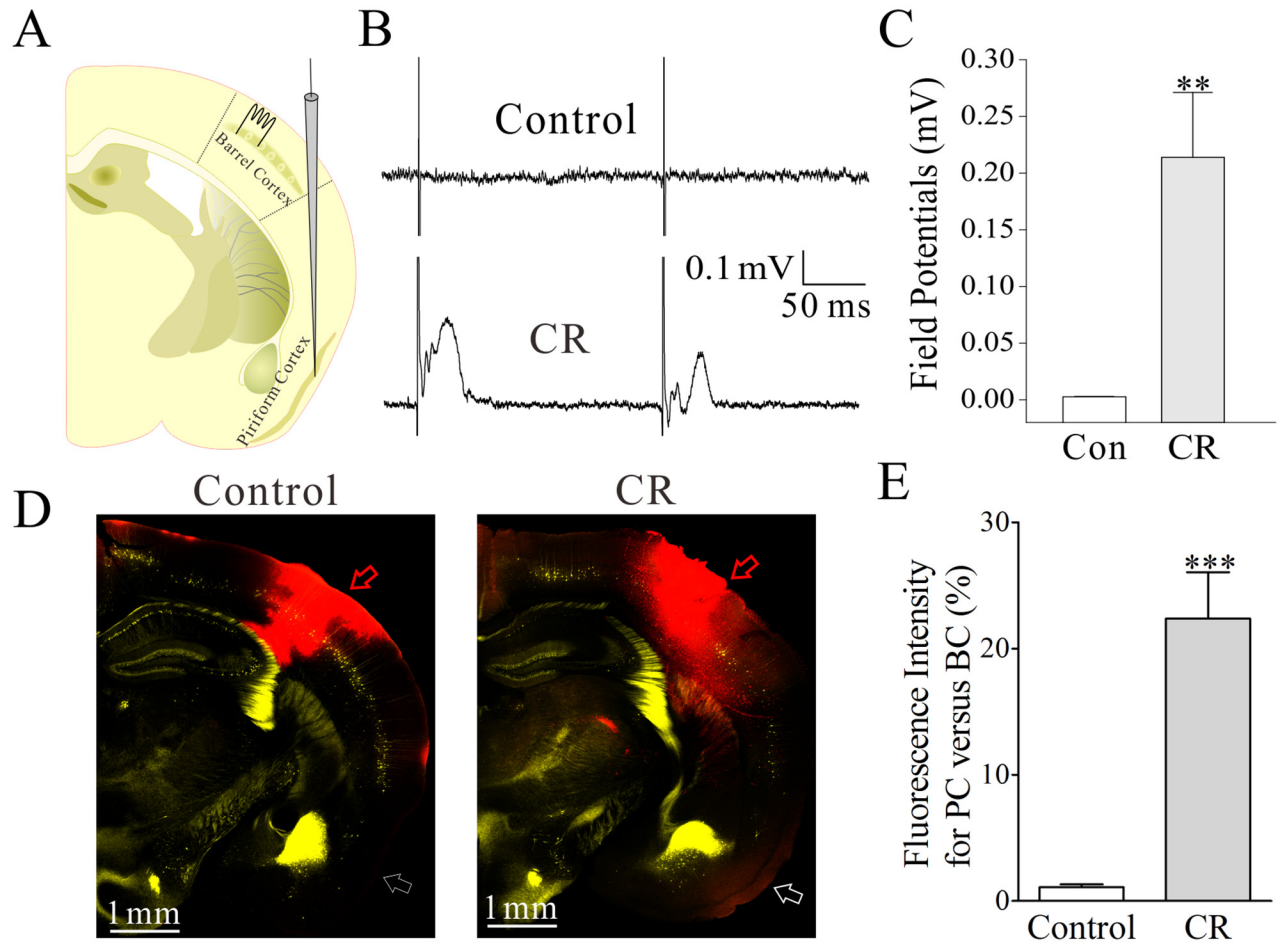
The barrel and piriform cortices are connected after their associative activation Structural connections among the cortical areas were traced by injecting pAAV-SynaptoTag-Cherry-GFP into the barrel cortex and seeing its presence in the piriform cortex. In the pAAV injection, the glass pipettes were positioned in the barrel cortex (-1.0 mm posterior to the bregma, 2.75 mm lateral to midline and 1.5 mm in depth). The *in vivo* neuronal activities were recorded by LFP at the piriform cortex while stimulating the barrel cortex. **(A)** shows LFP recording in the piriform cortex and electrical stimuli in the barrel cortex. **(B)** Top trace shows no LFP recorded in the piriform cortex from a control mouse. Bottom trace shows LFP in the piriform cortex recorded from a CR-formation mouse. **(C)** illustrates the comparison of LFPs recorded in the piriform cortex from CR-formation mice (n=3, gray bar) and controls (n=3, white). **(D)** Right panel shows neural tracing from the barrel cortex to the piriform cortex in a CR-formation mouse, in which an arrow indicates mCherry labeling in the piriform cortex. Left panel shows the neural tracing from the barrel cortex to the piriform cortex in a control mouse. An arrows indicates no fluorescent labeling in the piriform cortex. **(E)** shows the comparison of neural tracing in the piriform cortex from CR-formation mice (n=9, gray bar) and control (n=9, white bar), based on relative fluorescent intensity.

A connection from barrel to piriform cortices hypothetically drives piriform cortical neurons to be recruited as associative memory cells that encode whisker signal alongside odor signal and to be coordinated in the plasticity between glutamatergic and GABAergic neurons. By stimulating the barrel cortex electrocally *in vivo*, we are able to record the LFPs of the integrated synaptic signals and spikes in the piriform cortex from CR-formation mice (n=6), but not control mice (Figure 3A-3C, n=3). The functional connection from the barrel to piriform cortices is emerged after associative learning. The structural connections are traced by injecting pAAV-SynaptoTag-mCherry [36] into the barrel cortex. Compared with the neural tracing in control mice, mCherry is detected in the piriform cortex from CR-formation mice (Figure 3D-3E, n=9). The connections between barrel and piriform cortices may constitue the primary driving force to recruit piriform cortical neruons to be associative memory cells.

### Excitatory neurons in the piriform cortex are upregulated in CR-formation mice

The recruitment of the excitatory neurons in the piriform cortex to encode whisker signals may be caused by the upregulations of their excitatory synaptic inputs and spiking ability or the downregulation of their inhibitory synaptic inputs. We tested this hypothesis by analyzing YFP-labeled glutamatergic neurons in the piriform cortex from CR-formation versus control mice. The apical dendritic spines at the excitatory neurons in layer II~III of the piriform cortices were measured under confocal microscope to detect morphological changes in excitatory synapses. By recording the neurons in this area of the brain slices, we analyzed sEPSCs to assess excitatory synapse efficacy, spiking ability to merit neuronal active intrinsic properties and sIPSCs to evaluate inhibitory synaptic transmission [24, 34].

**Figure 4:**
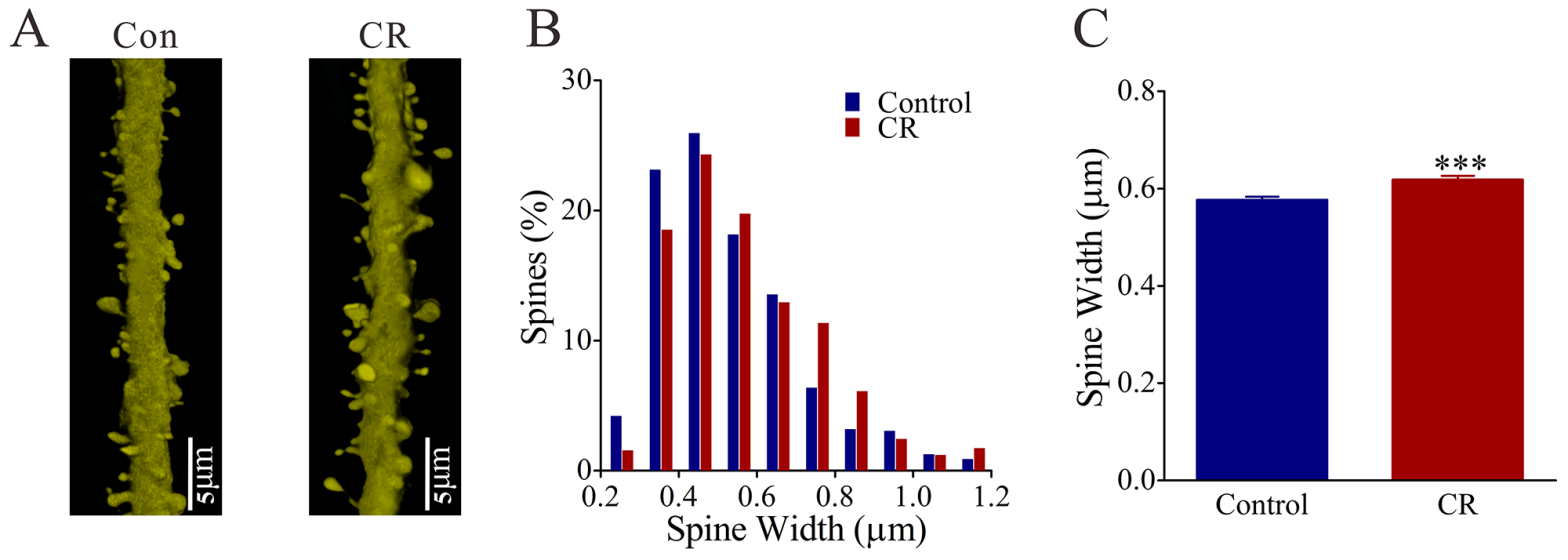
The head width of the spines on the glutamatergic neurons of the piriform cortex increases in the CR-formation mice **(A)** The spine head appears enlarged on the CR-formation dendrites (right panel) than controls (left). **(B)** illustrates the comparisons of spine widths from CR-formation (red bar, n=572 spines from four mice) and controls (blue, n=783 spines from four mice). **(C)** The spine heads tend to be large (asterisks, p<0.0001).

The size of spine head represents synapse efficacy since large heads are assumed to be the functional spines that form the synapses with axonal boutons [37]. The spine heads appear larger in CR-formation mice (right panel in Figure 4A) than controls (left). Spine head widths are 0.62±0.01 μm in CR-formation mice (red bars in Figure 4B-4C; n=572 spines from five mice) and 0.58±0.01 μm in controls (blue bars; n=782 spines from five mice, p<0.001; One-way ANOVA). Associative learning makes dendritic spines on glutamatergic neurons enlarged for synapse formation, which is consistent with a view that spine enlargement plays a role in memory [38].

**Figure 5:**
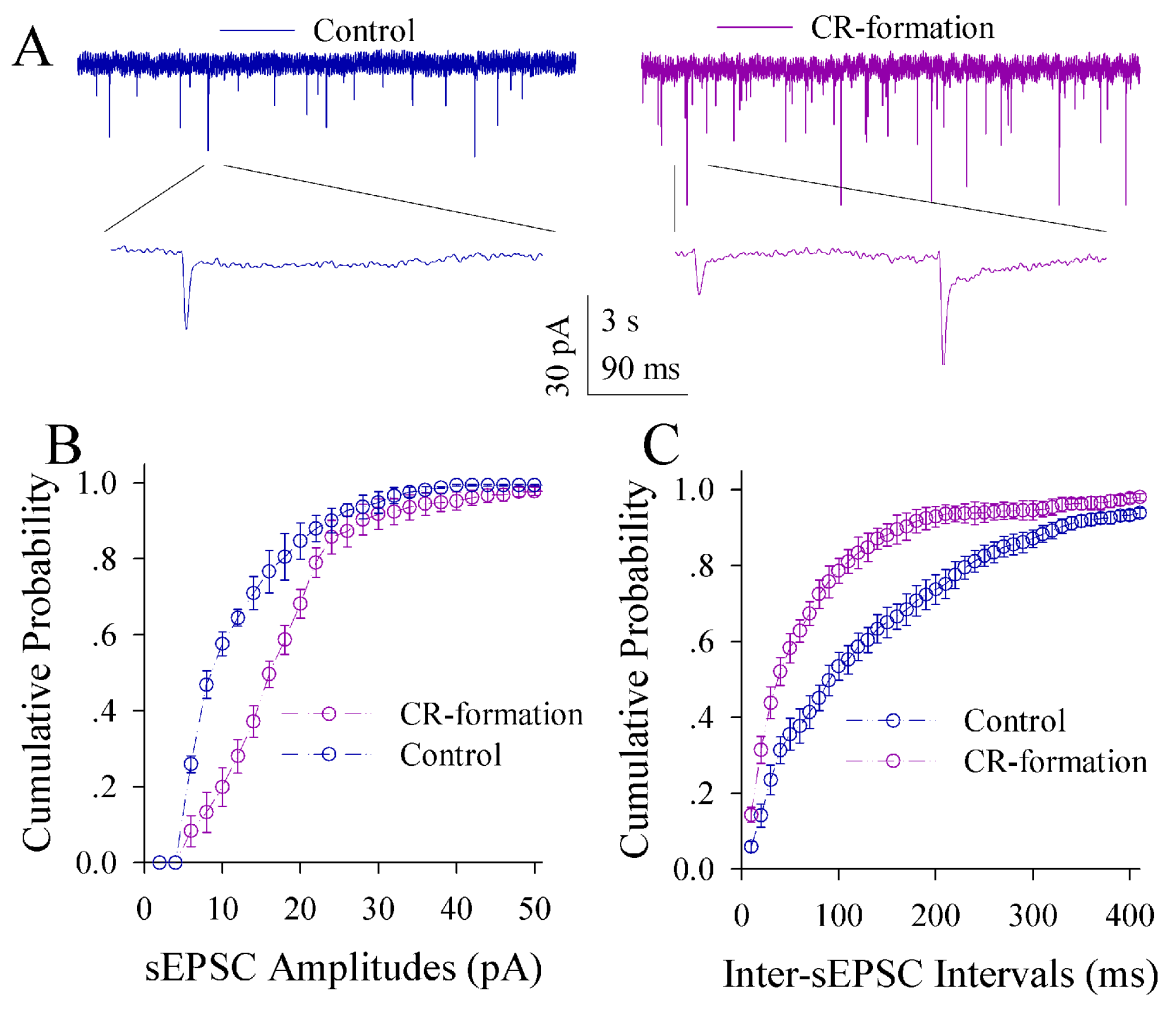
Excitatory synaptic transmission on the pyramidal neurons of the piriform cortices increases in CR-formation mice Spontaneous excitatory postsynaptic currents (sEPSC) were recorded on pyramidal neurons in cortical slices under voltage-clamp (holding potential at -70 mV) in the presence of 10 μM bicuculline. **(A)** illustrates sEPSCs recorded on a neuron from control mouse (blue trace in left panel) and CR-formation mouse (red in right). Bottom traces are the expanded waveforms selected from top traces. Calibration bars are 30 pA, 3 second (top) and 90 ms (bottom). **(B)** shows the cumulative probability of sEPSC amplitudes on the neurons from controls (blue symbols, n=15 neurons from nine mice) and CR-formation mice (red, n=15 neurons from nine mice). **(C)** illustrates the cumulative probability of inter-sEPSC intervals from controls (blue symbols, n=15 neurons from nine) and CR-formation mice (red, n=15 neurons from nine).

The influence of associative learning on excitatory synaptic transmission is illustrated in Figure 5. sEPSCs appear higher in CR-formation mice than controls (Figure 5A). Figure 5B illustrates cumulative probability versus sEPSC amplitude in CR-formation mice (n=15 cells from seven mice) and controls (n=15 cells from six mice). Figure 5C shows cumulative probability versus inter-sEPSC intervals from CR-formation mice (n=15 cells from seven mice) and controls (n=15 cells from six mice). Statistical analysis indicates that sEPSC amplitudes and frequencies (1/inter-sEPSC interval) increase in the excitatory neurons from CR-formation mice (p<0.01; One-way ANOVA). Associative learning upregulates the excitatory synaptic transmission at the glutamatergic neurons of the piriform cortices.

**Figure 6:**
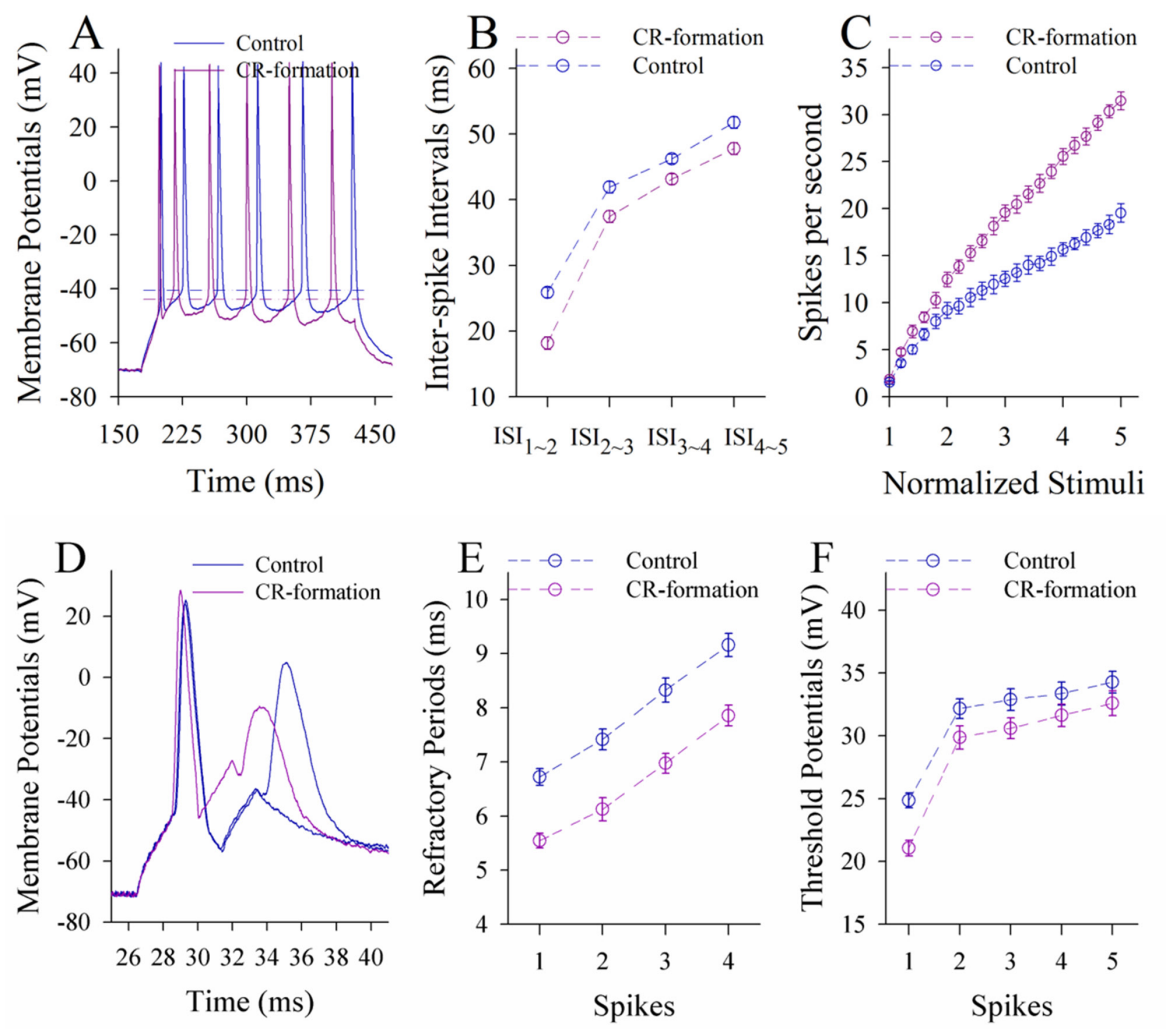
The ability to encode spikes on the pyramidal neurons of the piriform cortices increases in CR-formation mice The spikes were evoked by depolarization pulses under the current-clamp recording in glutamatergic neurons of the cortical slices. **(A)** Traces show depolarization-induced spikes on the neurons from control (blue trace) and CR-formation (red). **(B)** illustrates inter-spike intervals for spikes 1~2 up to spike 4~5 from controls (blue symbols, n=19 neurons from nine mice) and CR-formation (red, n=19 neurons from nine mice). **(C)** shows spikes per second vs. normalized stimuli (input-output) from controls (blue symbols, n=19) and CR-formation (reds, n=19 neurons from nine mice). **(D)** Traces illustrate the measurements of spike refractory periods on the neurons from control (blue trace) and CR-formation (red). **(E)** illustrates refractory periods versus spikes 1 to 4 from controls (blue symbols, n=19 neurons from nine mice) and CR-formation (reds, n=19 neurons from nine mice). **(F)** illustrates threshold potentials versus spikes 1 up to 5 from controls (blue symbols, n=19 neurons from nine mice) and CR-formation (reds, n=19 neurons from nine mice).

In terms of neuronal ability to convert excitatory inputs into spikes, the neurons in CR-formation mice appear higher ability to encode spikes (red trace in Figure 6A) than controls (blue). Figure 6B shows inter-spike interval (ISI) in the glutamatergic neurons from CR-formation mice (red symbols) and control (blue). ISI values in spikes 1~2 up to 4~5 are 18.16±0.96, 37.45±0.9, 43.15±0.79 and 47.78±0.92 in the neurons from CR-formation mice (n=19 cells from seven mice); and are 25.88±0.78, 41.93±0.87, 46.23±0.7 and 51.75±0.9 in controls (n=19 cells from six mice). ISI values for corresponding spikes in such two sources of neurons are different (p<0.01; One-way ANOVA). Moreover, in spikes versus normalized stimuli (Figure 6C), input-output curve in the neurons from CR-formation mice (red symbols) shifts left-high, compared with that in controls (blue). Associative learning raises the capability to convert excitatory inputs into spikes in the glutamatergic neurons of the piriform cortex.

Figure 6D-6F shows the changes of VGSC-mediated mechanisms, such as spike refractory periods (RP) and threshold potentials (Vts). Vts (Figure 6A) and RP (Figure 6D) appear lower in the neurons from CR-formation mice than controls. RP values for spikes 1 to 4 are 5.6±0.14, 6.12±0.2, 6.9±0.18 and 7.85±0.19 in the neurons from CR-formation mice (Figure 6E, n=19 cells from seven mice), and are 6.72±0.16, 7.42±0.19, 8.32±0.22 and 9.16±0.22 in controls (n=19 cells from six mice). Vts values for spikes 1~5 are 21.1±0.63, 29.86±0.9, 30.6±0.82, 31.6±0.9 and 32.5±0.9 in the neurons from CR-formation mice (Figure 6F; n=19 cells from seven mice), and are 24.86±0.6, 32.16±0.8, 32.87±0.88, 33.36±0.9 and 34.26±0.86 in controls (n=19 cells from six mice). RP and Vts for corresponding spikes in two sources of neurons are different (p<0.01; One-way ANOVA). Associative learning strengthens active intrinsic property in the glutamatergic neurons of the piriform cortex.

**Figure 7:**
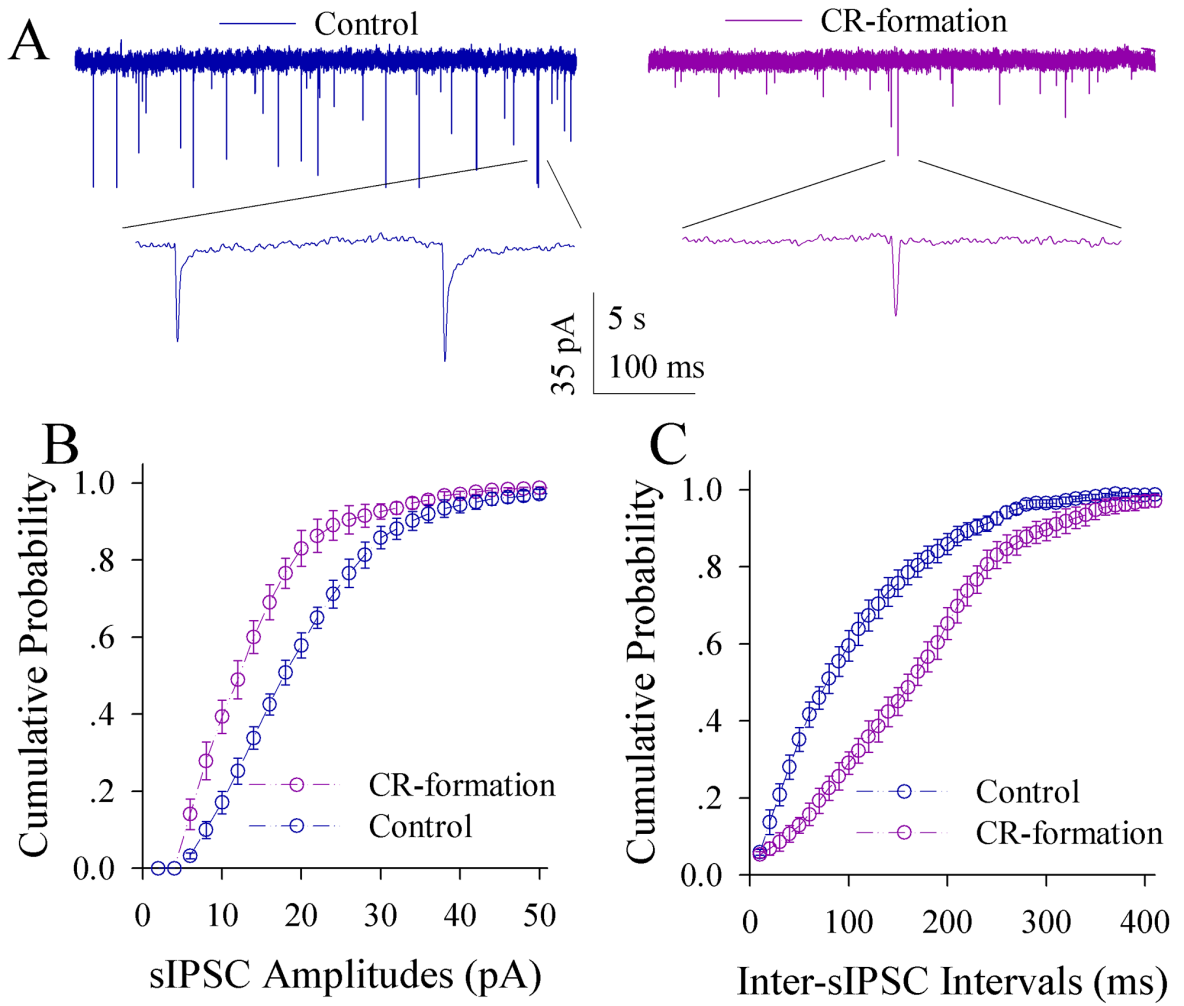
Inhibitory synaptic transmission on the pyramidal neurons of the piriform cortices decreases in CR-formation mice Spontaneous inhibitory postsynaptic currents (sIPSC) were recorded in glutamatergic neurons of cortical slices under voltage-clamp (holding potential at -65 mV) in the presence of 10 μM CNQX and 40 μM D-AP5. **(A)** Traces show sIPSCs recorded on the neurons from control (blue in left panel) and CR-formation (red in right). Bottom traces are the expanded waveforms from top traces. Calibration bars are 35 pA, 5 second (top) and 100 ms (bottom). **(B)** shows the cumulative probability of sIPSC amplitudes from controls (blue symbols, n=14 neurons from seven mice) and CR-formation (reds, n=14 neurons from seven mice). **(C)** illustrates the cumulative probability of inter-sIPSC intervals from controls (blue symbols, n=14 neurons from seven mice) and CR-formation (reds, n=14 neurons from seven mice).

The effect of associative learning on inhibitory synapse transmission in the glutamatergic neurons of the piriform cortex is illustrated in Figure 7. sIPSCs appear lower in CR-formation mice than controls (Figure 7A). Figure 7B illustrates cumulative probability versus sIPSC amplitudes in CR-formation mice (n=14 cells from six mice) and controls (n=14 cells from six mice). Figure 7C shows cumulative probability versus inter-sIPSC intervals in CR-formation mice (n=14 cells from six mice) and controls (n=14 cells from six mice). Statistical analyses demonstrate that sIPSC amplitude and frequency are significantly low in the glutamatergic neurons of CR-formation mice (p<0.01; One-way ANOVA). Associative learning makes inhibitory synaptic transmission weakened in the glutamatergic neurons of the piriform cortex.

Associative learning leads to the upregulation of excitatory synapses and encoding ability as well as the downregulation of GABAergic synaptic transmission on the glutamatergic neurons in the piriform cortex. These refinements may facilitate the recruitment of piriform cortical glutamatergic neurons to be associative memory cells.

### Plasticity at the inhibitory neurons of the piriform cortex in CR-formation mice

The processes, excitatory synaptic input and active intrinsic property of GFP-labeled GABAergic neurons in layer II~III of the piriform cortices were studied in CR-formation mice and controls. The branches of GABAergic neurons were counted to merit their receptive fields. sEPSCs were recorded to estimate excitatory synaptic function on these neurons. The capability to convert excitatory inputs into digital spikes was measured to assess their active intrinsic property [34].

**Figure 8:**
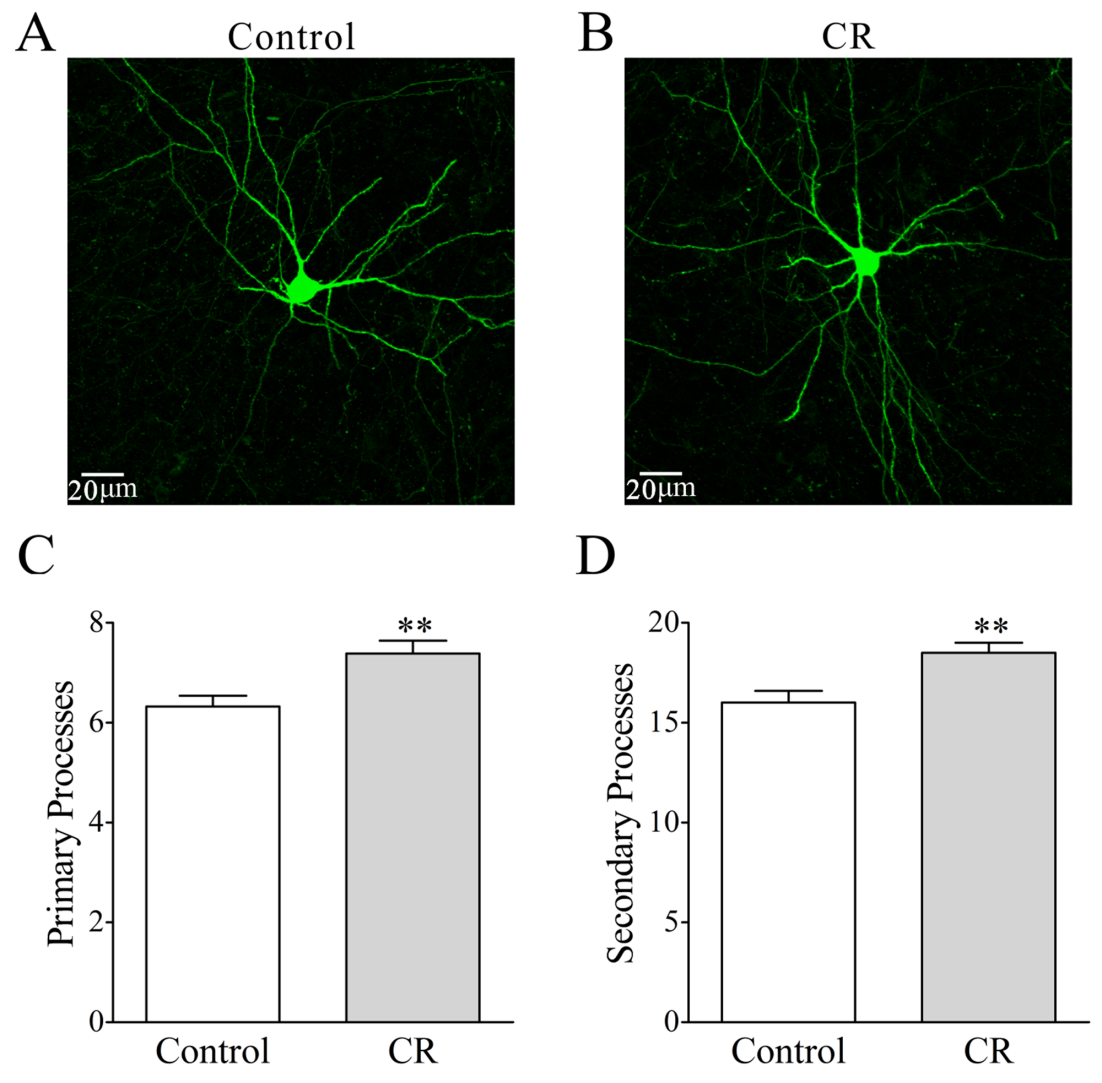
The processes of GABAergic neurons in the piriform cortices increase after pairing WS and OS **(A-B)** illustrates that process branches appear denser in CR-formation (B) than control (A). **(C)** Primary processes per GABAergic neuron are higher in CR-formation mice (gray bar, n=26 neurons from four mice) than controls (white, n=31 neurons from four mice; asterisk, p=0.002). **(D)** The secondary process branches per cell are higher in CR-formation (gray bar) than control mice (white bar, three asterisks, p=0.0026).

In the count of the processes of GFP-labeled GABAergic neurons, their branches appear denser in CR-formation mice (Figure 8B) than controls (8A). Primary processes per neuron are higher from CR-formation mice (7.4±0.25, n=26 cells from four mice) than controls (6.32±0.2, n=31 cells from four mice; p=0.002; One-way ANOVA; Figure 8C). The secondary processes per neuron are higher in CR-formation mice (18.5±0.5, n=26 cells) than controls (16±0.6, n=31 cells; p=0.0026; One-way ANOVA; Figure 8D). As the processes are locations for receiving synaptic inputs, GABAergic neurons have larger fields to receive excitatory inputs after associative memory onset.

**Figure 9:**
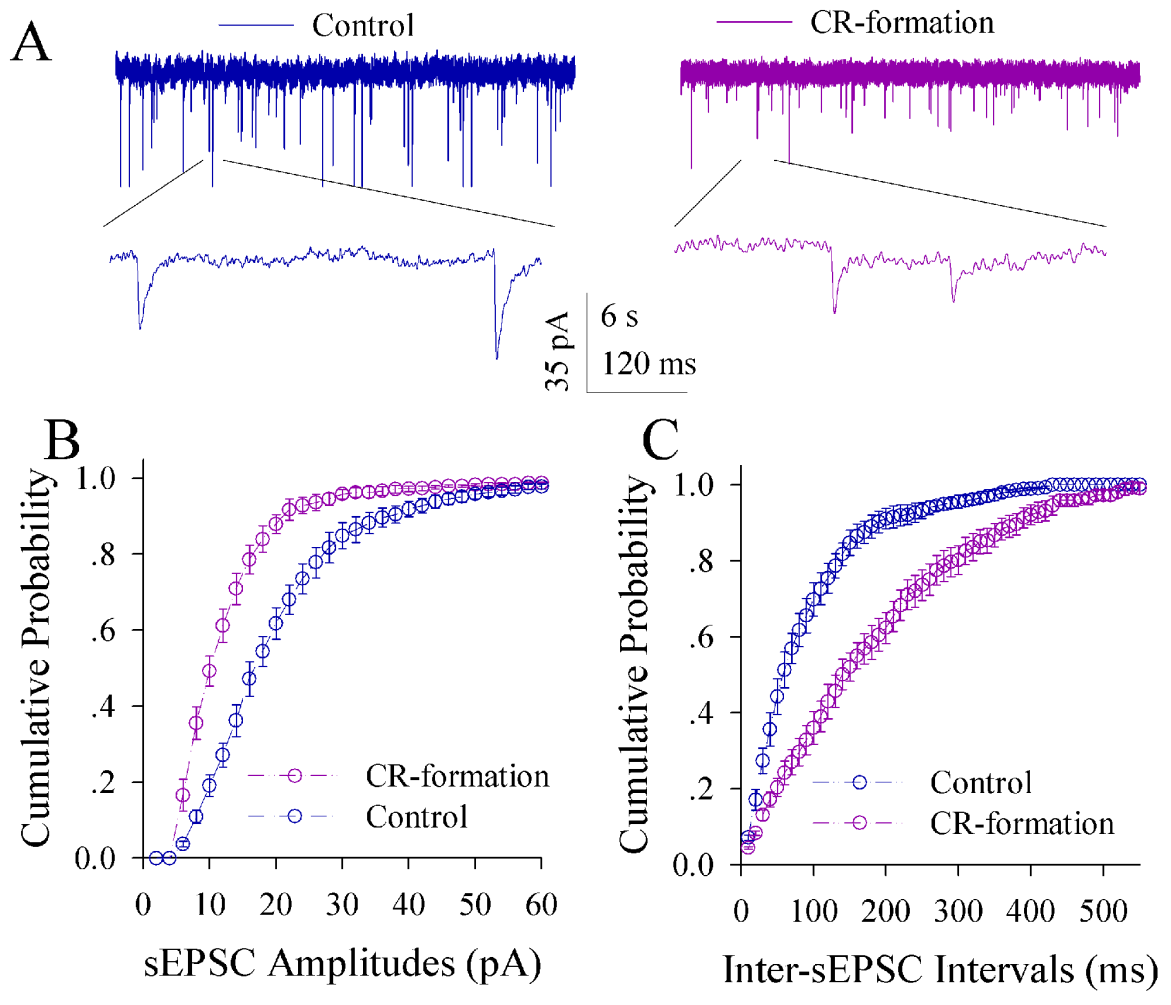
Excitatory synaptic transmission on the GABAergic neurons of the piriform cortices decreases in CR-formation mice Spontaneous excitatory postsynaptic currents (sEPSC) were recorded on the GFP-labeled GABAergic neurons in cortical slices under voltage-clamp (holding potential at -65 mV) in the presence of 10 μM bicuculline. **(A)** shows sEPSCs recorded on the neurons from control (blue in left panel) and CR-formation (red in right). Bottom traces are the expanded waveforms from top traces. Calibration bars are 35 pA, 6 second (top) and 120 ms (bottom). **(B)** illustrates the cumulative probability of sEPSC amplitude from controls (blue symbols, n=12 neurons from seven mice) and CR-formation (reds, n=12 neurons from seven mice). **(C)** illustrates the cumulative probability of inter-sEPSC intervals from control (blue symbols, n=12 neurons from seven mice) and CR-formation (reds, n=12 neurons from seven mice).

The influence of associative learning on excitatory synaptic transmission in GABAergic neurons is showed in Figure 9. sEPSCs appear lower in CR-formation mice than controls (Figure 9A). Figure 9B illustrates cumulative probability versus sEPSC amplitudes in CR-formation mice (n=12 cells from five mice) and control (n=12 cells from five mice). Figure 9C shows cumulative probability versus inter-sEPSC intervals in CR-formation mice (n=12 cells from five mice) and control (n=12 cells from five mice). sEPSC amplitude and frequency are lower in GABAergic neurons from CR-formation mice than controls (p<0.01; One-way ANOVA). Associative learning makes the GABAergic neurons in the piriform cortex to receive the reduced driving force from excitatory neurons.

**Figure 10:**
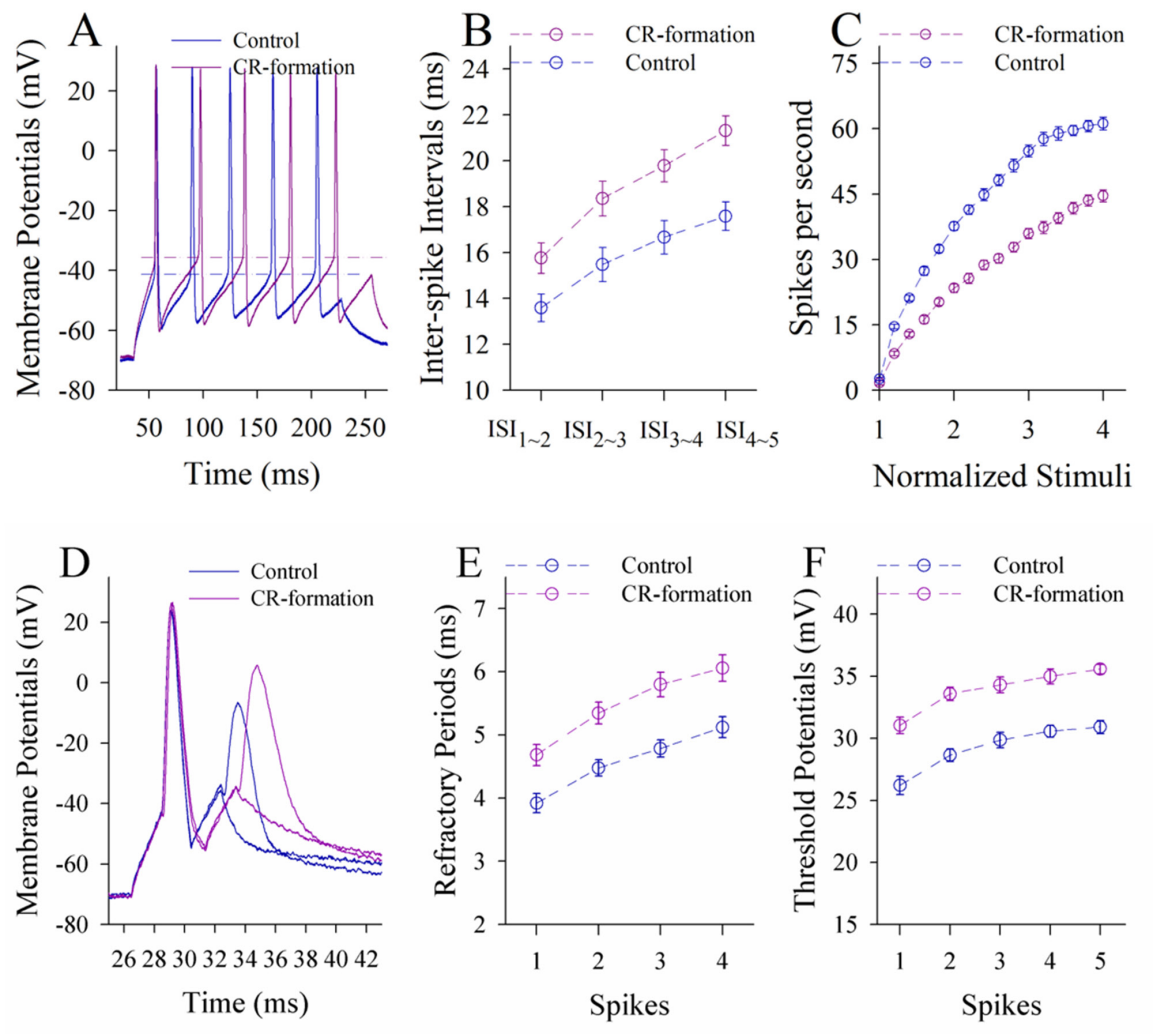
The ability to encoding spikes on the GABAergic neurons of the piriform cortices decreases in CR-formation mice Spikes were induced by depolarization pulses under current-clamp recording on the GABAergic neurons in cortical slice. **(A)** shows depolarization-induced spikes on the neurons from control (blue trace) and CR-formation (red). **(B)** shows inter-spike intervals for spikes 1~2 to 4~5 from controls (blue symbols, n=20 neurons from seven mice) and CR-formation (reds, n=20 neurons from seven mice). **(C)** shows spikes per second vs. normalized stimuli from controls (blue symbols, n=20) and CR-formation (reds, n=20 neurons from seven mice). **(D)** shows the measurements of spike refractory periods on the neurons from control (blue trace) and CR-formation (red). **(E)** shows refractory periods versus spikes 1 to 4 from controls (blue symbols, n=20 neurons from seven mice) and CR-formation (reds, n=20 neurons from seven mice). **(F)** shows threshold potentials versus spike 1~5 from control (blue symbols, n=20) and CR-formation (reds, n=20).

Figure 10A-10C illustrates the ability of GABAergic neurons to convert excitatory inputs into digital spikes. The neurons in CR-formation mice appear lower capability to encode spikes, compared with controls (Figure 10A). Figure 10B illustrates inter-spike intervals (ISI) in GABAergic neurons from CR-formation mice (red symbols) and controls (blues). ISI values for spikes 1~2 up to 4~5 are 15.76±0.66, 18.36±0.76, 19.78±0.7 and 21.3±0.64 in the neurons from CR-formation mice (n=20 cells from five mice); and are 13.6±0.6, 15.5±0.74, 16.67±0.73 and 17.58±0.62 in controls (n=20 cells from five mice). The ISI values for corresponding spikes in the neurons from CR-formation mice and controls are different (p<0.01; One-way ANOVA). In addition, the input-output curve in the neurons from CR-formation mice (red symbols in Figure 10C) shifts to right-low, compared with that in controls (blues). Associative learning attenuates the ability of GABAergic neurons to convert excitatory inputs into digital spikes.

Figure 10D-10F shows VGSC-mediated mechanisms at GABAergic neurons. Vts and RPs (Figure 10A, 10D) appear higher in the neurons from CR-formation mice than controls. RP values for spikes 1 up to 4 are 4.68±0.17, 5.35±0.18, 5.8±0.19 and 6.1±0.21 in the neurons from CR-formation mice (Figure 10E, n=20 cells from five mice), and are 3.92±0.15, 4.48±0.13, 4.78±0.14 and 5.12±0.17 in controls (n=20 cells from five mice). Vts values for spikes 1~5 are 31.04±0.66, 33.58±0.53, 34.3±0.65, 34.98±0.6 and 35.56±0.4 in the neurons from CR-formation mice (Figure 10F; n=20 cells from five mice), and are 26.2±0.73, 28.65±0.5, 29.85±0.63, 30.57±0.48 and 30.9±0.52 in controls (n=20 cells from five mice). RP and Vts values for corresponding spikes in two sources of the neurons are different (p<0.01; One-way ANOVA). Associative learning attenuates active intrinsic property in GABAergic neurons of the piriform cortex.

Associative learning downregulates excitatory synaptic driving force and spiking ability in the GABAergic neurons of the piriform cortex, despite upregulating the receptive field of the excitatory synaptic inputs (homeostasis among subcellular compartments; [39]). The downregulated spiking ability in GABAergic neurons and inhibitory synaptic transmission facilitate the recruitment of the excitatory neurons to be associative memory cells in the piriform cortex after associative learning.

### Plasticity of mutual innervations between excitatory and inhibitory neurons in CR-formation mice

**Figure 11:**
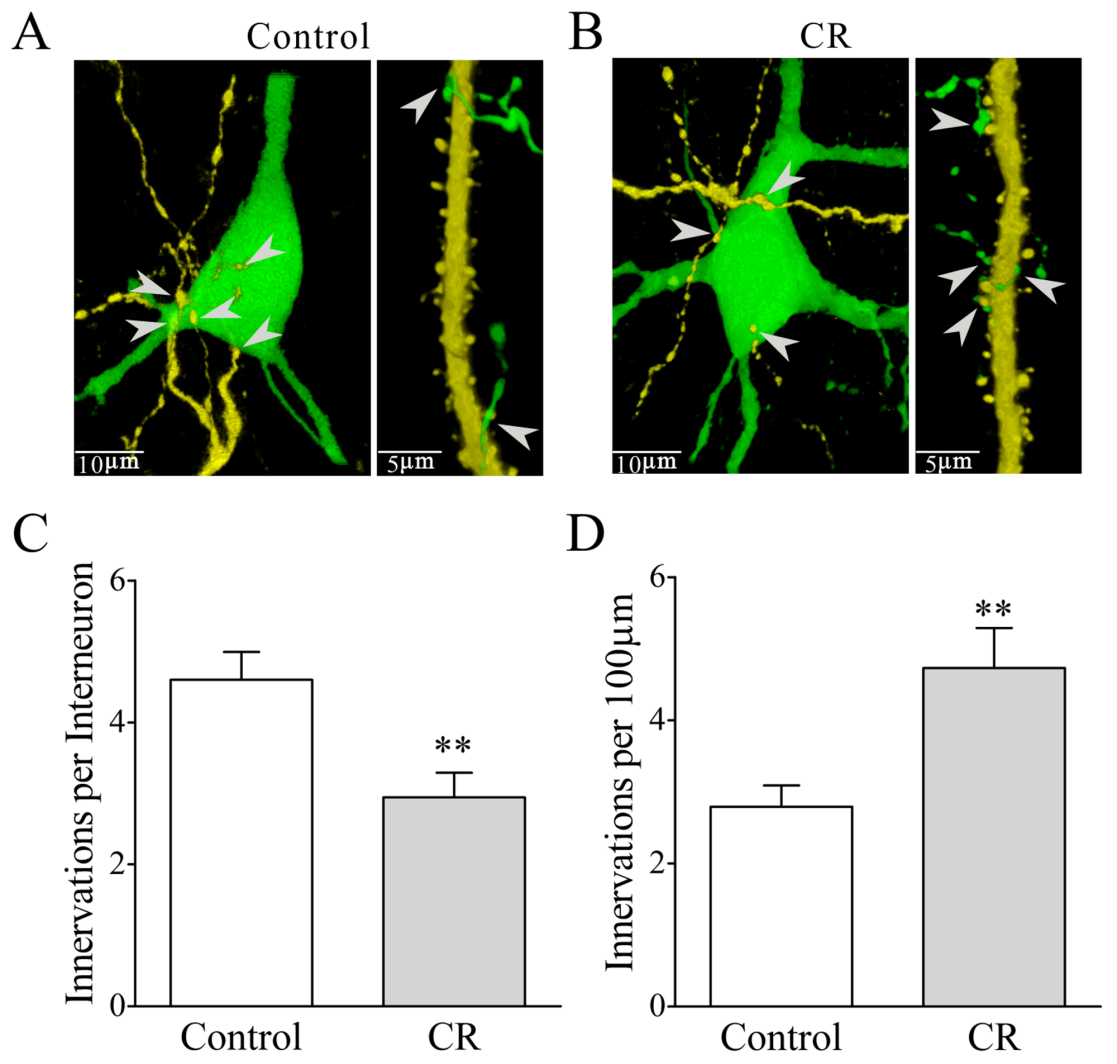
Mutual innervations between excitatory and inhibitory neurons are upregulated in associative learning The neuronal imaging scanned under a confocal microscope was conducted in the mice whose glutamatergic neurons were genetically labeled by YFP and GABAergic neurons were labeled by GFP. **(A-B)** illustrate the mutual innervations between glutamatergic and GABAergic neurons in control (A) and CR-formation (B). YFP-labeled axonal terminals on each GFP-labeled GABAergic neuron (left panel) and GFP-labeled axonal terminals per 100 μm of YFP-labeled apical dendrites on glutamatergic neurons (right) are showed and analyzed. White arrows indicate their axon terminations. **(C)** shows YFP-labeled axonal terminals on each GABAergic neuron from control (white bar, n=20) and CR-formation (gray bar, p=0.0035, n=18). **(D)** shows GFP-labeled axonal terminals per 100 μm of YFP-labeled apical dendrites in controls (white bar, n=23) and CR-formation (gray, p=0.0025, n=18).

To mutual innervations between glutamatergic and GABAergic neurons, we counted YFP-labeled axonal boutons on each GFP-labeled GABAergic neuron and GFP-labeled axonal boutons per 100 μm of YFP-labeled apical dendrites on each glutamatergic neuron. As illustrated in Figure 11A-11B, their mutual innervations appear altered in CR-formation mice. YFP-labeled axonal boutons on each GABAergic cell are 4.6±0.39 in controls (white bar in Figure 11C, n=20 cells from four mice) and 2.94±0.35 in CR-formation mice (gray bar, p<0.01, n=20 cells from four mice; One-way ANOVA). GFP-labeled axon boutons per 100 μm of YFP-labeled apical dendrites are 2.79±0.3 in control mice (white bar in Figure 11D, n=20 cells from four mice) and 4.7±0.56 in CR-formation mice (gray, p=0.025, n=23 cells from four mice; One-way ANOVA). Associative learning upregulates the innervation of inhibitory neurons onto excitatory neurons and downregulates the innervation of excitatory neurons onto inhibitory neurons.

### microRNA profile in the piriform cortices of CR-formation mice versus controls

What are molecular mechanisms for the coordinated plasticity between excitatory and inhibitory neurons, the axon projection from the barrel to piriform cortices as well as the recruitment of associative memory cells after associative learning? High throughput sequencing was used to detect the differential expression of the molecules, in which the microRNA profiles were analyzed in the piriform cortices from CR-formation mice and controls. Table 1 illustrates the differential expressions of microRNAs, i.e., some microRNAs are downregulated and others are upregulated. Their downregulation allows the enhanced expressions of their target genes and proteins, or vice versa. Based on genes’ functions from references in www.ncbi.nlm.nih.gov/gene/ and www.amigo.geneontology.org, the alternation of microRNA expression in CR-formation mice is associated to upregulating glutamatergic synapse formation, process growth and synaptic function, as well as downregulating GABAergic synaptic transmission. In addition to revealing new microRNAs that are involved in memory formation (Table 1), their roles in regulating neurons and synapses support the indications from the study of neuron-specific plasticity and memory cell recruitment (Figures 3-11).

**Table 1:** MicroRNA's expression and their roles in associative learning

Name of microRNA	MicroRNA expression in CR-formation versus control	Predicted target genes	Functions of target genes
	Piriform cortex		
mmu-miR-342-5p	↓↓	Neto1 Elfn1 Fgfr2 Gria1 Grin1 Lrrtm2 Nlgn3	
		Gria1	
		Elfn1	Excitatory synapses
mmu-miR-324-5p	↑↑	Grin1Lrrtm2Nlgn3Fgfr2	
mmu-miR-5128	↑	Fgfr2	
mmu-miR-150-5p	↑	Csnk1dNlgn2	
mmu-miR-3072-3p	↑	Slc32a1	
mmu-miR-150-5p	↑	Nlgn2	
mmu-miR-324-5p	↑↑	Slc32a1Nrxn1	Inhibitory synapses
mmu-miR-23b-3p	↑↑	Nrxn1	
mmu-miR-133a-3p	↑↑	Iqsec3	
mmu-miR-874-5p	↑↑	Ywhaq	
mmu-miR-345-5p	↑	Gabra4Rgma	synapse formation or neuron branching
mmu-miR-149-3p	↑↑	Rgma	
mmu-miR-324-5p	↑↑	Ttbk1	
mmu-miR-133a-3p	↑↑	Dyrk2	Axon guidance and cytoskeleton
mmu-miR-3072-3p	↑	Mark2	
mmu-miR-874-5p	↑↑	Gmip	
mmu-miR-345-5p	↑	Rgma	Spines and dendrites
mmu-miR-324-5p	↑↑	Ttbk1	
mmu-miR-149-5p	↑↑	RhoaRgma	
mmu-miR-149-3p	↑↑	Rhoa	
mmu-miR-3072-3p	↑	Wnk2	Channels
mmu-miR-149-5p	↑↑	↑↑	
mmu-miR-150-5p	↑	Csnk1d	Neurite outgrowth
mmu-miR-133a-3p	↑↑	Dyrk2	
mmu-miR-149-3p	↑↑	Rgma	
mmu-miR-133a-3p	↑↑	Dyrk2	Neuron proliferation and differentiation

↑: log_2_(CR-formation/Control) <1; ↑↑: 1≦log_2_(CR-formation/Naïve control) < 2; ↑↑↑: log_2_(CR-formation/Control) ≧2.

## DISCUSSION

**Figure 12:**
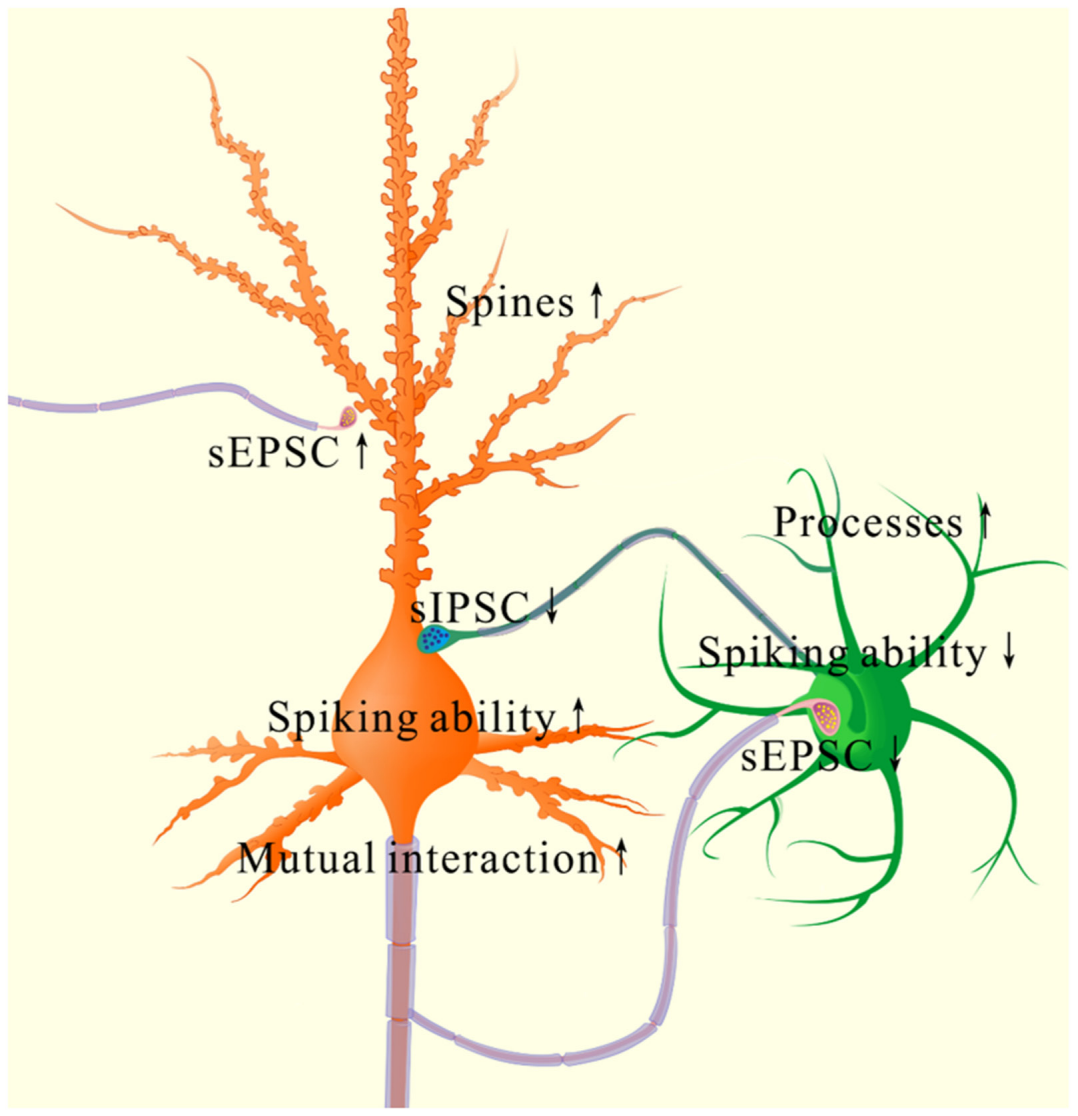
The coordinated plasticity between excitatory and inhibitory neurons in the piriform cortex for memory formation In excitatory neurons (orange), their spines are enlarged, their excitatory synapse function is enhanced, their spiking ability is raised as well as their function controlled by inhibitory synapses is lowered. These changes drive the excitatory neurons to an optimal state for their recruitment and refinement to store newly learnt whisker signal. However, their innervations from GABAergic axons is increased, such that the excitatory neurons are not overexcited. In inhibitory neurons (green), their reception from excitatory synaptic transmission/innervations, their intrinsic property and synaptic outputs are lowered. These changes facilitate the functional recruitment and refinement of their downstream cells for information storage. However, their processes are enriched, which keeps a homeostasis of GABAergic neurons by coordinating different subcellular compartments.

Associative learning by pairing whisker and odor stimulations leads to whisker-induced olfaction responses (Figure 1). In mice that show this cross-modal associative memory, piriform cortical neurons become able to process the newly learned whisker signal alongside the innate odor signal for their integrations and joint storages as well as to encode such associated signals differently for their distinguishable retrieval, i.e., associative memory cells (Figure 2). The recruitment of these associative memory cells for this cross-modal memory may be driven by new synapse innervations from the barrel cortex to piriform cortex (Figure 3). To these associative memory cells in the piriform cortex, excitatory synapses and spiking ability in glutamatergic neurons are upregulated (Figures 4-6) and inhibitory synaptic transmission is downregulated (Figure 7). GABAergic neurons decrease in their intrinsic properties and reception from glutamatergic synapses (Figures 8-10). All of these changes facilitate the recruitment and refinement of piriform cortical neurons to be associative memory cells. Moreover, the plasticity of mutual innervations between excitatory and inhibitory neurons (Figure 11) tends to keep the homeostasis of local neuronal networks (Figure 12).

Information storage is presumably achieved by plasticity in dendritic spines and synapses [40–45] and in neuronal digital spikes [46, 47]. This belief is based on the observations that neural plasticity occurs in their innate input signals (non-associative learning) and neuronal units are analyzed without cell-specific merit. In our studies of cross-modal associative memory, i.e., whisker-induced olfaction response, piriform cortical neurons *in vivo* learn to encode the newly learned whisker signal from the barrel cortex alongside their innate odor signal (Figures 2-3). The recruitment of associative memory cells in the piriform cortex to encode new whisker signal may also be a cellular mechanism widely used in the cerebral cortex for storing new information. In this cross-modal associative memory, excitatory synapses and spiking capability rise at the glutamatergic neurons and lower at the GABAergic neurons. Their coordination may drive the piriform cortex to an optimal state for the functional recruitment and refinement of network neurons to memorize the newly learned signal and the innate signal associatively.

Associative learning evokes a coordinated plasticity at subcellular compartments of glutamatergic neurons. The upregulation in their dendritic spines and excitatory synaptic transmission (Figures 4-5) and the downregulation of their GABAergic synaptic reception (Figure 7) will coordinately strengthen the excitatory driving force to initiate digital spikes at these excitatory neurons. In addition, their ability to encode spikes rises (Figure 6). These changes in subcellular compartments make glutamatergic excitatory neurons to have the increased ability and precision of encoding digital spikes [48–50], such that more excitatory neurons are recruited and more active neurons are refined to encode the storage and retrieval of the associated signals. The recruitments and refinements of more excitatory neurons to be associative memory cells will boost their capabilities to activate their downstream neurons in the cortical circuits for memory presentation [22, 25]. Our results provide the cellular bases for the assumption that the synchronization of neuronal activity facilitates memory formation [51]. It is noteworthy that the upregulated excitatory synapses and the downregulated inhibitory synapses may not occur in all of the synapses on glutamatergic neurons to make their over-excitation. We suggest that the recruitment and refinement of piriform cortical neurons as associative memory cells are based on new synapse innervations from the axon projection of barrel cortical neurons. This suggestion is supported by our observations that new synapse innervations lead to increased sEPSC frequency (Figures 3 and 5) and spine head enlargement is consistent with raised sEPSC amplitudes (Figures 4 and 5). In addition, when certain GABAergic neurons receive new synapse innervations from barrel cortical neurons to be recruited as associative memory cells [32], they are functionally downregulated (Figures 8-10) and in turn attenuate the function of GABAergic synapses on glutamatergic neurons (Figure 7). In other words, the refinement and plasticity in piriform cortical neurons occur in subcellular compartments relevant to new synapse innervation and associative memory cell for the storage and retrieval of associated signals.

To the role of GABAergic neurons and synapses in associative memory, our results indicate the decreases in their functions driven by excitatory inputs (Figure 9), their capabilities to code digital spikes (Figure 11) and their inhibitions to downstream neurons (Figure 7). These changes coordinately facilitate the recruitment and refinement of their innervated cortical neurons to be associative memory cells for information storage. Our study by analyzing subcellular compartments of GABAergic neurons supports a notion about the disinhibition of neural circuits for fear memory [23]. On the other hand, the dendrites of GABAergic cells and their axon terminations onto excitatory neurons increase after associative memory (Figures 8 and 11). The upregulations in these subcellular compartments of GABAergic neurons may be used to strengthen their functions and to maintain local neuronal networks not being overexcited in memory formation. To the glutamatergic neurons in CR-formation mice, an upregulated GABAergic axon innervation and a downregulated GABA input function indicate cellular homeostasis maintained by opposite changes in function and structure. To the GABAergic neurons in CR-formation mice, the downregulations of somatic excitatory inputs and spike encoding as well as the upregulations of their dendrites and axon terminals are another example that homeostatic neuronal function is maintained through the coordination of subcellular compartments [39]. These forms of cellular homeostasis are presumably used for long-term maintenance of memory formation.

To the mechanism for recruiting associative memory cells, initiating plasticity in subcellular compartments and strengthening neuronal innervations in the piriform cortex, we hypothesize that the associated activations of the barrel cortex by whisker signal and the piriform cortex by odor signal induce their mutual connection. The new axon projection and synapse innervation lead to neuron refinement and associative memory cell recruitment. These axons from the barrel cortices may innervate OS-responsive neurons in the piriform cortex to make them encoding WS and OS (associative memory neurons), and/or innervate sensory silent neurons to activate them encoding WS only (new memory neurons). The neurons in response to the WS and OS present the historical association and the recognition of these associated signals. The barrel cortical projected axons and innervations onto piriform cortical neurons may also strengthen their sensitivity to the odor signal in response to environment alerts. This hypothesis remains to be examined by using neuron-specific tracing. In fact, the mutual projection of new axons and the innervation of new synapses have been showed by neural tracing between the piriform and barrel cortices [30–32]. In addition to granting our findings, the mutual innervation, synapse formation and neuron plasticity in both piriform and barrel cortices after associative learning indicate that these cortices share similar mechanism for memory formation. Mutual innervations between the piriform and barrel cortices make the terms of “conditioned” and “unconditioned” stimulations not being present. Either whisker signal or odor signal is able to induce cross-modal associative memory and responses in the reciprocal manner [25, 30, 32].

miRNA profile in the piriform cortices from CR-formation mice was analyzed by sequencing miRNAs to reveal molecular mechanism underlying memory-related changes (Figures 3-11), such as the coordinated neuronal plasticity, the new synaptic innervation from the barrel cortex to the piriform cortex and the associative memory cell recruitment. The increased level of miRNAs knocks down their target messenger RNAs, or vice versa (Table 1). The altered microRNA expression in Table 1 facilitates axon growth, synapse formation and excitatory synaptic function as well as impede inhibitory synaptic function. In addition to consistent results from functional, morphological and molecular approaches, our data reveal that certain miRNAs (Table 1) are involved in memory formation. Therefore, we hypothesize a testable diagram for the mechanisms of memory formation. The associations of whisker and odor signals activate the piriform cortex and the barrel cortex. Their elevated activities of these sensory cortices thorough their intensive action potentials trigger epigenetic events, such as alternations in the expression of certain miRNAs. These epigenetic changes regulate the expression levels of the targeted mRNAs and the translated proteins, which instigate axon projection, synapse formation and neuron plasticity. This hypothesis is being tested by injecting the antagomirs of miRNAs into these cortices, in which the antagomirs of miRNA-3245p and miRNA-133a appear to attenuate cross-modal associative memory through lowering associative memory cell recruitment and new pathway-specific synapse innervation [32, 52].

Associative memory is essential for cognitive processes, such as logical reasoning, associative thinking and comparison. These cognitions require the associated storage and retrieval of various paired signals and/or events in the different groups of associative memory cells and their integrations [25]. To fulfill cognitions, these groups of associative memory cells work jointly through the retrievals of the stored signals in sequential and multi-grade manners or through a common signal in these various paired signals for their integrations. In this regard, the newly wired circuits among different brain regions and the newly formed synapses in neural circuits are essential to the communication of associative memory cells for cognitions. We propose characteristics of associative memory cells in sensory cortices (i.e., primary associative memory cells). In addition to the innate innervation from a specific sensory input, they morphologically receive new synapse innervations from other sensory cortices that innately encode different sensory inputs being associated for their primary integration and storage. They functionally encode multiple associated signals including their innate signal and newly learnt signals from external environments. Their axons project and innervate onto the neurons in downstream brain areas relevant to behavior, cognition and emotion to make these downstream neurons responding to these associated signals (i.e., secondary associative memory cells) and to initiate memory presentation. The number of the recruited associative memory cells and their strengthened refinements are proportional to memory strength and maintenance. Their recruitments are controlled by epigenetics-regulated genes and proteins relevant to memory via a chain reaction of intensive spikes and microRNA expression alteration. Cognitive processes, such as associative thinking, logical reasoning, imagination, computation and so on, activate primary and secondary associative memory cells to induce their axon projections and synapse innervations onto the neurons in cognitive brain regions for the integrations and storages of these endogenous signals, leading to cognition-related memories. The recruitment of associative memory cells and their plasticity influence physiological and pathological processes related to memory involvement [21, 25]. In the storage and retrieval of associated signals, the working principle for associative memory cells is based on their receptions to innate and new synapse inputs, their abilities to convert synaptic analogue signals into digital spikes for encoding associated signals and their capacities to output spikes for driving behavior-, cognition- and emotion-related brain areas in memory presentation. Therefore, the synapse inputs to associative memory cells determine the specificity of memory contents. The activity power and plasticity at associative memory cells as well as at their input and output partners may set up the strengths of information storage and memory presentation.

## MATERIALS AND METHODS

All experiments were performed based on guidelines by Administration Office of Laboratory Animals at Beijing China. Experiment protocols were approved by Institutional Animal Care Unit Committee in Administration Office of Laboratory Animals at Beijing China (B10831).

### Mouse model of associative memory

C57 Thy1-YFP/GAD67-GFP mice [34] were used, in which their glutamatergic neurons were genetically labeled by yellow fluorescent protein (YFP) and GABAergic neurons were labeled by green fluorescent protein (GFP). By using these mice whose cerebral cortices include YFP-labeled glutamatergic neurons and GFP-labeled GABAergic neurons, we are able to study neural plasticity in their structures and functions clearly in the cell-specific manner as well as to analyze their subcellular compartments and mutual interactions.

Mice in postnatal day 20 were set into two groups that were trained by pairing mechanic whisker stimulus (WS) with odorant stimulus (OS, butyl acetate toward the noses) and by unpairing these stimuli as control (WS and OS with five minutes in intervals), respectively [21, 22, 24, 31]. Butyl acetate was 99.99% in analytical purity. The pairing or unpairing of the WS and OS was given by a multiple-sensory modal stimulator (ZL201410499466), in which the intensity, time and interval of OS and WS were precisely set (video one in [31]). The OS intensity was sufficient to induce the responses of olfactory bulb neurons, and the WS intensity was enough to induce whiskers’ fluctuation after WS ended [31]. Each mouse was trained twenty seconds in each time, five times per day with two hours of intervals, and ten days in a home-made cage. The cares were paid attention including no stressful experimental condition and circadian disturbance to mice that possessed normal whisking and symmetric whiskers [21, 24, 31, 53].

The onsets of whisker-induced olfaction responses were examined after the trainings for ten days. During the test, the mice were placed in the central arm of the T-maze [35], their assigned whiskers were stimulated (similar to the training paradigm), and their motions toward other two arms that include an object coated with butyl acetate versus an object without butyl acetate coating were monitored (Figure 1). A few of principles used in this test are given below. 1) The distances between the objects and central zone in the T-maze were set by moving two objects symmetrically away from the central zone to the positions where the mice moved into either of two arms just to be an equal chance (i.e., no statistical difference in moving into either of two arms) before training, in control and without whisker stimulus. In other words, the mice reached to the central zone under these conditions were not able to smell butyl acetate from the butyl acetate-coated object [35]. This belief was based on a fact that the odorant diffusion in air and its dilution by air caused odor concentration to be gradually reduced with the distance. This principle allowed to measure olfactory sensitivity based on the distance from the animals to odorants, and to read out the minimal concentration of odorants just before the animals responded to the source of the odorants correctly, i.e., the threshold of olfactory sensitivity [35]. After pairing the WS and OS, the olfaction responses (i.e., mice moved toward or away from the odorants) by stimulating their whiskers were due presumably to that these mice smelled the odorants. This method to test the presence of whisker-induced olfaction response was based on the belief that the mice became able to smell the training odorant (butyl acetate) by whisker stimuli after WS/OS-pairing. In other words, the whisker stimulation to WS/OS-paired mice induced their recall of this trained odorant while seeing the increase of their olfactory sensitivity triggered by stimulating whiskers. 2) Two objects were randomly placed in either arm to prevent their memory to previous reactions toward either left or right as well as to make sure their motions based on the odorant smelling. 3) The rates that the mice correctly selected the arms were calculated from the ten times of the test for each mouse, and averaged from the groups of the mice. 4) As butyl acetate was an aversive stimulus to the mice, they intended to move into an arm without butyl acetate-coated object after OS and WS association (please see video one). The onset of whisker-induced olfaction responses is warranted if the percentage of selecting the arm including the object without coating butyl acetate increases significantly after OS/WS-associated training.

Cares were taken in the followings. T-maze was cleaned by 70% ethanol and then water-wet papers before each trail to remove any odors adhering on T-maze walls. The fresh blocks were used for each trail to maintain the consistency of odor concentration. Experiments were done in a 60 m^2^ room with the good and constant ventilations. The movement of the experimenters in the room was restricted to prevent making odor plume and noise. The mice moving into either arm above 30 centimeters and staying above 5 seconds were counted as their entrance [35]. The “assigned whiskers” were long whiskers (such as arcs 1~2) on the same side and same rows that were assigned for the training and the testing by mechanical whisker stimuli. We did not trim short whiskers since the whisker trimming raised barrel cortical excitability [34], which might affect the conditioning responses.

### Electrophysiological recordings *in vivo*

After the completion of the behavioral training for 48 hours, mice were anesthetized by the intraperitoneal injection of urethane (1.5 g/kg). During operations, the anesthetic depth was set as lack of reflexes in pinch withdrawal and eyelid blinking. The body temperature was maintained by the heating blanket electronically controlled at 37°C. The location of the piriform cortex for *in vivo* recording was judged based on mouse brain mapping [54], which was confirmed by histological reconstructions after each experiment. The craniotomy (2 mm in diameter) was made on the skull above the center of the piriform cortex. The *in vivo* recordings at the piriform cortex were positioned to be 0.34~0.58 mm posterior to the bregma, 3.25~3.5 mm lateral to midline and 4.75~5.0 mm in the depth. The anesthetic depth of the mice during *in vivo* electrophysiological study was set at their moderate reflex in pinch withdrawal and eyelid blinking, as well as their whiskers in responses to the test stimulation, i.e., a light anesthesia.

Local field potentials (LFP) were recorded in layers II~III of piriform cortices by using glass pipettes that contained a standard pipette solution (150 mM NaCl, 3.5 mM KCl and 5 mM HEPES). The resistance of recording pipettes was 5~7 MΩ. Electrical signals were inputted to an AxoClamp-2B amplifier and pClamp 10 system (Axon Instrument Inc. CA USA), in which the Clampex and the Clampfit were applied for data acquisition and analysis, respectively. Electrical signals were digitized at 20 kHz and filtered by low-pass at 0.5 KHz. In data analyses, the 1~100Hz band-pass filter and second order Savitzky-Golay filter were used to isolate LFP signals. LFP waveforms were complex and variable. Individual LFP events induced by whisker or odor stimulations lasted about 10 ms with a sharp response in low frequency (<10 Hz). LFP peaks were the most consistent parameter to be quantified [55, 56]. The differences between LFP peak and baseline were measured and averaged to present stimulus-evoked LFP amplitude. LFP frequency was one over the averaged intervals of LFP signals in the stimulations.

In electrophysiological recordings *in vivo*, the tests of odorant stimulus and whisker deflection were given to mice of control versus whisker-induced olfaction response, respectively. In consistence with the OS and WS that were used in behavioral training in terms of patterns, intensities and frequencies, an odor-test pulse toward the noses or mechanical pulses to the assigned whiskers on the contralateral side of recorded cortical areas were used to evoke cellular responses in piriform cortices. In sequential stimulation to olfaction and whiskers, stimulus pattern was pair-pulse (OS versus WS or WS versus OS) and their intervals were 60 seconds. Neuronal responses in piriform cortices to the WS and the OS indicated the storage of these associative signals. The differences of responsive amplitude and frequency to the WS and the OS in the neurons indicated their recognitions to associated signals during information retrieval. In the experiment of the stimuli to the barrel cortex and the recording at the piriform cortex, the electrical stimulus pulses were 0.2 ms and an intensity that induced 80% of maximal responses in CR-formation mice, which was also used for control mice.

### Neural tracing

Structural connections among cortical areas were traced by injecting pAAV-SynaptoTag-Cherry-GFP (a gift from Dr. Tom Sudhof) into the barrel cortex and by detecting its presence in the piriform cortex from C57 Thy1-YFP mice. The working principle of this AAV was that Synapsin-I promoter initiates the expression of EGFP-synaptobrevin-2 in presynaptic boutons and terminals as well as the expression of mCherry in entire neurons, especially axons [36]. In pAAV injection, glass pipettes were positioned in the barrel cortex (-1.0 mm posterior to the bregma, 2.75 mm lateral to mid-line and 1.5 mm in depth), based on mouse brain mapping [54]. As the peak of YFP emission wavelength was 525 nm, we scanned it by setting the optical grate at 540 nm and the excitation wavelength was 488 nm [21, 24, 34]. Functional connections were examined by stimulating the barrel cortex and recording LFP in the piriform cortex.

### Brain slices and neurons

Cortical slices (400 μm) in coronal section were prepared in mice with whisker-induced olfaction response and control. They were anesthetized by isoflurane inhaling and decapitated by guillotine. Slices were cut with Vibratome in oxygenated (95%O_2_/5%CO_2_) artificial cerebrospinal fluid (ACSF), in which the concentrations (mM) of chemicals were 124 NaCl, 3 KCl, 1.2 NaH_2_PO_4_, 26 NaHCO_3_, 0.5 CaCl_2_, 4 MgSO_4_, 10 dextrose and 5 HEPES (pH 7.35 and 4 °C). Slices were held in oxygenated ACSF (124 NaCl, 3 KCl, 1.2 NaH_2_PO_4_, 26 NaHCO_3_, 2.4 CaCl_2_, 1.3 MgSO_4_, 10 dextrose, and 5 HEPES, pH 7.35) at 25°C for 2 hours. A slice was placed to a submersion chamber (Warner RC-26G) that was perfused with ACSF oxygenated at 31°C for whole-cell recording [39, 57–59]. Chemical reagents were from Sigma.

Electrophysiological recordings on GFP-labeled GABAergic neurons and YFP-labeled glutamate neurons in layers II-III of piriform cortices were conducted under a DIC-fluorescent microscope (Nikon FN-E600, Japan). The wavelength at 488 nm excited the fluorescence of GFP-labeled neurons, and the wavelength at 575 nm excited the fluorescence of YFP-labeled neurons. GABAergic neurons demonstrated fast spiking with less adaptation in spike amplitude and frequency, typical properties for the interneurons [48, 49, 60, 61]). Glutamatergic neurons showed the pyramidal shape of their somata and the adaptation of spike amplitude and frequency. Cortical slices were the sections including the barrels correspondent to projections from longer whiskers that were stimulated by pairing the WS and the OS during the mouse training.

### Whole-cell recordings for neuronal functions in piriform cortices

Neurons were recorded by a MultiClamp-700B amplifier under the voltage-clamp for their synaptic activities and the current-clamp for their active intrinsic properties. Electrical signals were inputted into a pClamp-10 (Axon Instrument Inc, CA USA) for data acquisition and analysis. The output bandwidth in this amplifier was set at 3 kHz. The pipette solution for recording excitatory events included (mM and pH 7.35) 150 K-gluconate, 5 NaCl, 5 HEPES, 0.4 EGTA, 4 Mg-ATP, 0.5 Tris-GTP, and 5 phosphocreatine [62–64]. The solution for investigating inhibitory synapses contained (mM and pH 7.35) 130 K-gluconate, 20 KCl, 5 NaCl, 5 HEPES, 0.5 EGTA, 4 Mg-ATP, 0.5 Tris–GTP and 5 phosphocreatine [65, 66]. Pipette solutions were freshly made and filtered (0.1 μm). Their osmolarity was 295~305 mOsmol and pipette resistance was 5~6 MΩ.

The functions of GABAergic neurons were assessed based on their active intrinsic properties and inhibitory outputs [67]. Inhibitory outputs were assessed by recording spontaneous inhibitory postsynaptic currents (sIPSC) under voltage-clamp on pyramidal neurons in the presence of 10 μM 6-Cyano-7-nitroquinoxaline-2,3-(1H,4H)-dione (CNQX) and 40 μM D-amino-5-phosphonovanolenic acid (D-AP5) in ACSF to block ionotropic glutamatergic receptors. 10 μM bicuculline was washed onto slices at the end of experiments to examine whether synaptic responses were mediated by GABA_A_R. It blocked sIPSCs in our experiment. It is noteworthy that pipette solutions with a high concentration of chloride ions makes reversal potential to be -42 mV. sIPSCs are inward when the membrane holding potential is at -65 mV [66, 68, 69].

The functions of excitatory neurons were evaluated based on their active intrinsic properties and excitatory outputs [67]. Excitatory outputs were assessed by recording spontaneous excitatory postsynaptic currents (sEPSC) on GABAergic neurons or pyramidal neurons in presence of 10 μM bicuculline in the ACSF to block ionotropic GABAergic receptors [50, 67]. 10 μM CNQX and 40 μM D-AP5 were added into ACSF perfused onto slices at the end of experiments to examine whether synaptic responses were mediated by glutamate receptor. They blocked EPSCs in our experiments.

Action potentials at these cortical neurons were induced by injecting depolarization pulses, whose intensity and duration were set based on the aim of experimental merits, such as inter-spike interval, input-output, threshold potential and refractory period. The ability to convert excitatory inputs into digital spikes was evaluated by inter-spike intervals (ISI) when depolarization pulses (200 ms in duration and threshold for 10 ms pulse-induced spike in intensity) were given. This ability was also evaluated by input-outputs (spikes versus normalized stimuli), in which stimulus intensities were step-increasing by 10% normalized stimulations. As the excitability of different cells was variable these step-raised depolarization pulses were given based on their normalization. The base value of stimulus intensity for this normalization at each neuron was the threshold of depolarization pulse (300 ms in duration) for evoking a single spike [70]. Neuronal active intrinsic properties included spike threshold potentials (Vts) and absolute refractory periods (ARP). Vts were the voltages of firing spikes, which were the points that dV/dt values show suddenly huge increases in the initiation phase of spikes [48, 57, 62]. ARPs were measured by injecting paired-depolarization pulses (3 ms) into these neurons after each spike. By changing inter-pulse intervals, we defined ARP to be a duration from a complete spike to its subsequent spike at 50% firing probability [71, 72].

Data were analyzed if recorded neurons had resting membrane potentials negatively more than -60 mV, and action potential amplitudes more than 90 mV. The criteria for the acceptance of each experiment also included less than 5% changes in resting membrane potential, spike magnitude, and input resistance throughout each experiment. Series and input resistances in all of the neurons were monitored by injecting hyperpolarization pulse (5 mV/50 ms), and calculated by voltage pulses versus instantaneous and steady-state currents, in which there was no change in such values between control versus CR-formation. To assess the effect of associative learning on neuronal spikes and synaptic activity, we measured sEPSC, sIPSC, ISI, ARP and Vts in the neurons of brain slices from control and CR-formation mice. Their values were presented as mean±SE.

### Cellular morphological imaging in piriform cortex

The structures of YFP-labeled glutamatergic neurons and GFP-labeled GABAergic neurons were examined by the confocal laser scanning microscope. CR-formation mice and controls were anesthetized by intraperitoneal injections of sodium pentobarbital (40 mg/kg), as well as were perfused by 4% paraformaldehyde in 0.1 M phosphate buffer solution (PBS) into the left ventricle/aorta until their bodies were rigid. The brains were quickly isolated from these mice and fixed in 4% paraformaldehyde PBS for additional 24 hours. The cortical tissues were sliced in the coronal section of the piriform cortex at 80 μm by a Vibratome. These sections were rinsed by PBS for 3 times, air-dried and cover-slipped. Images for YFP-labeled and GFP-neurons in the piriform cortices were photographed under laser scanning confocal microscopy (Nikon A1R plus, Japan). Despite overlaps of GFP and YFP in their spectra, the peaks of their emission wavelength are 510 nm and 525 nm, respectively. In this regard, we scanned GFP by setting the optical grate at 505-515 nm and YFP by setting the optical grate at 535-545 nm, respectively, to un-mix their images.

The structures of these neurons were analyzed by public software ImageJ (version 1.47; National Institute of Health, USA). As the brain tissues were sliced in series sections, the counting and analysis in cell structures could be done at least from two sections. The analyzed sections were chosen in a manner of one section from every two in order to prevent the influence of cells that crossed the neighboring sections. In the analyses of the dendrites, the primary processes (branches from soma) and the secondary processes (branches from primaries) of glutamatergic and GABAergic neurons were measured in each of sections. In the glutamatergic neurons, their apical dendrites were analyzed [73]. Their spines were protrusions extended from the dendrites, which were counted as spines per 100 μm dendrite and their width [34]. In terms of morphological interaction between the different types of the neurons in the piriform cortices from the mice, we analyzed mutual innervations between these neurons by counting contacts of presynaptic boutons with postsynaptic neurons.

### Statistical analyses

The paired t-test was used in the comparisons of the experimental data before and after associative learning, as well as the neuronal responses to whisker stimulus and odorant stimulus in each of the mice, such as Figures 1 and 2. One-way ANOVA was applied to make the statistical comparisons in the changes of neuronal activities and morphological quantification between control and associative learning groups, such as Figure 3 up to Figure 11.

### An analysis of microRNA by microRNA-sequencing

Total RNAs were isolated from the samples of piriform cortices in control and CR-formation mice by using TRIzol reagent (Life Technologies, Grand Island, USA) based on manufacturer guidelines. The fractionation and preparation of smaller RNAs (18-30 nts) were done by using the Protocol of Small RNA Sample Preparation (Illunina) for high throughput sequencing. The sequencing was done by using Illumina HiSeq 2000 system (Illumina, CA, USA).

Small RNAs in HiSeq deep sequencing included miRNA, siRNA, piRNA, rRNA, tRNA, snRNA, snoRNA, repeat associated sRNA and degraded tags in exons or introns. By comparing our data with those in databases and picking up their overlaps on genome locations, small RNAs were annotated into different categories. Those, which could not be annotated, would be used to predict novel miRNA by a self-developed software Mireap (BGI-Shenzhen, China).

50nt sequence tags from Hiseq sequencing went through data cleaning analyses. This analysis would get rid of low quality tags and 5′ adaptor contaminants from 50nt tags to get credible clean tags. The length distributions of these clean tags as well as the common and specific sequences in these samples from control versus CR-formation were summarized. The standard analysis annotated clean tags into different categories and took those which could not be annotated to any category to predict novel miRNA and potential known miRNA. Finally, the target prediction for miRNA as well as the GO enrichment and the KEGG pathway for target genes were analyzed [24].
